# Trust, technocracy, and the public servant’s bargain: The evolving role of Canadian health leaders post-COVID

**DOI:** 10.1177/08404704251320301

**Published:** 2025-02-21

**Authors:** Jared Wesley, Samuel Goertz

**Affiliations:** 1 170234 University of Alberta, Edmonton, Alberta, Canada

## Abstract

Public servants are central in helping Canadians navigate public health crises. Before, during, and after the COVID-19 pandemic, these professionals have been essential to implementing widespread government interventions, sometimes amid significant public scrutiny. These experiences highlight the delicate balance public health officials maintain in a democracy: providing expert advice to cabinet to define the public good and implementing decisions to help preserve public health. Notwithstanding varying scopes for autonomous decision-making, chief medical officers of health aid elected officials in weighing tradeoffs in the pursuit of communal objectives, not by dictating them but by enabling informed decision-making. In recent years, there have been calls for public health officials to substitute their judgement for that of elected officials in issuing directives. This article explores the role of public health officials as public servants and the perils of these officials misunderstanding their roles which may undermine the effectiveness and legitimacy of policy decisions.

## Introduction

From those in frontline service delivery to those in government departments, public servants found themselves at the forefront of the COVID-19 pandemic response. The global health crisis and ensuing economic downturn touched everyone’s lives directly, with long-lasting and disparate effects on our health, relationships, and livelihoods. Governments responded to these changes by intervening in ‘people’s’ lives to an unprecedented extent. The ensuing policy changes, from tax increases to public health restrictions to travel bans, faced significant pushback from certain segments of the public, though the vast majority of people remained compliant. All the while, Canadians have participated in dozens of local, provincial, and federal elections in the last few years, with many appearing more heated than the last.

Political discourse has been particularly charged when it comes to public health policy, with effects that may linger far beyond COVID-19. Throughout the pandemic, most Canadians placed greater trust in public health officials and local healthcare providers over elected politicians.^1^ To the extent that there was unease with government restrictions, Canadians tended to saddle politicians with more of the blame. In this atmosphere, there have been some calls for Public Health Officials (PHOs) to exercise more decision-making authority and work in greater isolation from the influence of elected officials.^2-4^ Given their role as appointed leaders tasked with advising governments and recommending policy options, many PHOs, such as chief medical officers of health, are considered “public servants.” Our focus in this paper is on those particular public health leaders who directly interact with and advise elected officials. Their experiences exemplify the tensions between the technical and political aspects of policy-making in health and other areas.^5,6^

Public servants play an important but circumscribed role in Canadian democracy: providing fearless advice and loyally implementing decisions made by cabinet. In return for security of employment and the anonymity that comes from working within the bureaucracy, public servants provide elected officials with evidence-informed options, recommendations, and a commitment to implement their agenda. Ministers are held accountable for all decisions made under their portfolios by elected members of the legislature who derive their power from voters. This “public servants bargain” has served Canadians well throughout the past two centuries.^7^ Empowering unelected bureaucrats to make decisions outside of this structure amounts to technocracy: a form of governance that, on the surface, provides depoliticized decision-making based on expertise, but that removes the ability of voters to hold policy-makers to account and weakens the all-important relationship between the elected and appointed sides of government.

In this article, we explore the role of PHOs in the policy-making process, arguing for restraint when it comes to expanding their powers beyond those exercised by cabinet. The discussion presented here is particularly relevant to health leaders who navigate the intersections of public trust, health policy, and politics. We explain the tradeoffs between different models of health policy-making as a means of assisting these public servants in navigating the politicized world of public administration.

## Policy-making in a time of declining trust

The COVID-19 pandemic has deepened fissures between certain segments of the public and government, eroding trust in policy-makers.^8^ Approval ratings for federal leaders are at fifty-year lows^9^ and trust in governments generally is at an ebb. Between 2020 and 2022, trust in elected officials dropped 18 percentage points among Canadians.^10^ Those experiencing health problems or financial hardship were among the most disaffected.^11^

While confidence rose slightly in 2023 and appears to be recovering some of the losses associated with the pandemic,^12^ the rise of populist parties at the federal and provincial levels suggests anti-elite sentiments remain high. To date, this populist shift has been less pronounced in Canada than elsewhere, yet its steady rise dates back prior to the pandemic.^13^

All of this serves to undermine the public’s trust in government decision-making and judgement, particularly that of elected officials. Thus, the argument for decision-making power to be devolved from elected officials to bureaucrats, particularly in highly complex and specialized areas like public health, appears to be a strong one. This has surfaced age-old tensions over the proper place of public servants in Canadian democracy. On one hand, addressing crises and day-to-day problems requires some level of expertise and potentially unpalatable responses. With higher levels of trust and legitimacy among the public and protection from political pressures, it is possible that non-partisan public servants could implement more effective public health measures. On the other hand, the impacts of public health decisions are so wide-ranging that vesting power in unelected bureaucrats seems less than democratic, ultimately hindering the effectiveness of such decisions.

Further, while trust in politicians and government is lower overall, PHOs, and public servants of all kinds, have not been immune to this trend. The percentage of Canadians who reported a great deal of trust in PHOs declined from 59% to 37% between March 2020 and March 2022.^14^ So while PHOs played an important role during the pandemic, their increased visibility and involvement in contentious decisions subjected them to public scrutiny and eroded trust.

Research from the comparable American context documents that visibility, particularly for chief medical officers of health, led to a sharp increase in harassment, personal attacks, and public backlash towards health leaders.^15-17^ This isn’t to say that PHOs deserved any mistreatment or skepticism, merely that their increasingly prominent and politicized role created and amplified tensions in their relationship with the public. Eroded trust had clear consequences for the effectiveness of public health measures. Statistics Canada data from 2020 shows a strong negative relationship between trust in PHOs and willingness to follow public health measures and get vaccinated.^18^

With a clear mandate to advance public health and operate freely from political pressure, it might seem that separating public health officials from elected governments would enhance the effectiveness of public health measures. However, a closer examination of their role as public servants within our government system reveals the significant risks of such separation. These risks not only threaten democratic legitimacy but also undermine the overall effectiveness of public health initiatives.

## Technocracy and democratic legitimacy

When people argue that public health officials, particularly chief medical officers of health, should have greater independence and decision-making power, they are essentially advocating for a shift toward technocracy, or “government by experts.”^19^ Technocracy is premised on the belief that political ideologies and the drive to remain popular produce poor public policy outcomes. Instead, the argument goes, policy-making should be entrusted to leaders and administrators chosen based on their technical skills and knowledge. Policy decisions should be grounded in scientific evidence and rational analysis, rather than political motivations. For example, some feel that a more technocratic approach to the COVID-19 pandemic would have reduced the spread of the disease and prevented more deaths than one predicated on placating the electorate.

While the pandemic provides a case study, similar tensions have arisen in other public health scenarios, such as vaccine distribution campaigns, the drug-poisoning crisis, and climate-related health emergencies. These examples illustrate the widespread challenge of balancing technocratic expertise with public trust and democratic legitimacy.

As attractive as it may appear on the surface, particularly during divisive political times, technocracy conflicts with the norms of Canadian democracy in two ways. First, it advances the notion that policy-makers can and should be chosen based on merit rather than election. Second, technocracy holds that the public good can or should be defined purely by scientific evidence and expertise. Both premises undermine the legitimacy of government decision-making in Canada. They are also unlikely to lead to better public policy.

Contrary to the wishes of technocratic proponents, empowering PHOs with more decision-making authority would likely result in *more* politicization of their roles, not less. Knowing that these officials would be vested with such autonomy, governments would be unlikely to handle appointments through a non-partisan process. Given that their decisions would be beyond the purview of government itself, cabinet would wish to select people who share their outlook on public policy. Appointments would be based as much on ideological alignment and partisan loyalty as merit, undermining one of the key advantages of technocracy in the first place. Here, it is worth remembering: in a technocracy, the public does not get to choose the technocrats. Rather, these officials are chosen by those with political power who then place them in positions with little public accountability.

Calls for technocracy in the health sector may seem expedient given the need for high levels of expertise and impartiality. However, these calls often reflect a deeper problem of depleted trust in government. The COVID-19 pandemic highlighted the critical role of PHOs but also showcased the dangers of their guidance and decisions being perceived as politically motivated. This was seen in the decline of trust in public servants cited earlier, which, while remaining higher than politicians, paralelled the drop in confidence in elected officials. In extreme cases, increasing decision-making power for PHOs could inflame accusations from certain segments of the population that feel experts exert too much influence without representing the will of the broader public.^20^

Every day public servants make many decisions that affect the lives of Canadians without being subject to direct oversight by elected officials. A degree of devolved decision-making is essential to efficient government operations. However, this devolution is delimited by legislation; public servants cannot act outside the legal boundaries set by cabinet and individual ministers.

While informed by the fearless advice provided by public servants, the direction of government is set within what cabinet views as the public good. Moreover, ministers are individually and collectively responsible to the legislature for the actions taken by public servants under their areas of purview. Thus, all government decisions are made and checked by elected officials who are accountable to the electorate. Technocracy interrupts this system of responsible government, providing unelected officials with the power to both define and pursue the public good without being accountable to Canadians.

## Technocracy and public health efficacy

Ultimately, lower levels of trust and accountability can erode a government’s *civic license* to pursue policy objectives. Civic license refers to the willingness of citizens to accept government decisions and make personal sacrifices on behalf of the broader society.^21^ It is an essential ingredient in policy success, particularly as it relates to public health. Where civic license does not exist, norms and rules can be challenged and, without higher levels of enforcement, this can lead to a vicious cycle where breaking rules becomes normalized.

Democratic decision-making forces governments to remain attuned to the public’s willingness to comply with their policies. To critics, this means popular appeasement takes precedence over scientific necessity. Governments may not pursue necessary public health measures for fear it may lose them votes in the next election. There is merit to this criticism. However, the same dynamics may encourage elected officials to engage in the necessary work of persuading the public to abide by important directives. Governing politicians must build civic license for their actions at risk of losing power. Technocrats tend to have fewer direct incentives in this regard, making them more likely to rely on enforcement to ensure compliance.

Technocrats are also isolated from the whole-of-government policy-making that occurs at the cabinet table. This weakness means that an autonomous chief medical officer of health is less able or likely to fully consider the broader social, political, and economic implications of their decisions. Whether by lack of understanding, resources, or the necessity of building consensus with other policy-makers, an independent chief medical officer of health could be poorly positioned to make well-informed, durable choices. In some cases, this could mean issuing directives that lack the necessary support and enforcement to implement. At worst, this could mean making decisions that conflict with those made in other parts of government, putting the public in a position of having to choose between competing sets of rules and guidelines.

## Revisiting the public servant’s bargain

The “public servant’s bargain” is fundamental to Canadian governance and underscores the interdependent relationship between elected officials and the bureaucracy. Public servants, including many PHOs, are not tasked with determining the public good or charting the path to achieve it. Instead, their role is to provide expert advice to the government and to do the critical work of carrying out government directives.

The space that public servants occupy in Canada is vital but it is also limited. The public servant’s bargain is crucial because it ensures that advice is given without fear or favour and that actions are taken within a democratic framework that maintains public trust. It is not the public servant’s role to hold the government accountable; that function is reserved for members of the legislative branch whose collective confidence cabinet must maintain. Rather, public servants provide advice to government, offering independent analyses of likely outcomes of pursuing various policy directions.

This system offers distinct advantages. It provides the basis for evidence-informed decision-making, as PHOs s can focus on their expertise, free from political pressures. Politicians are also better positioned to sell policy measures to the public, ensuring broader acceptance and compliance. Indeed, technocrats, no matter how skilled, cannot bypass this essential relationship between the elected and the electorate. Public servants ultimately rely on politicians to ensure that measures are not only implemented but also supported by the public, a critical element of their effectiveness and durability.

During the pandemic, some PHOs may have felt that political interference hindered their ability to act decisively and effectively. However, their effectiveness depends on weaving together the technical and political aspects of policy. The decline in trust in public health officials during the pandemic was less severe than in politicians. Yet, it appeared linked, demonstrating that confidence in health officials and policy must be upheld through cooperation between public servants and elected officials. They are mutually dependent and the more each side can support one another and exercise their separate responsibilities in a coordinated manner, the more effective government will be in serving Canadians.

## Conclusion

Although public servant expertise is essential to effective political decision-making, the success of policies also depends on public acceptance, which elected officials are uniquely positioned to secure. As Canada continues to navigate public health and public policy challenges, it is essential to uphold the established roles within government. For health leaders, this means actively engaging with elected officials to support evidence-informed decision-making, while fostering public trust in the policies they are tasked with implementing. Health leaders are also powerfully positioned to correct misinformation and champion the importance of robust methodology when assessing evidence, both when speaking with elected officials and the public. For elected officials, this means understanding and upholding their end of the public servant’s bargain as well, by soliciting non-partisan advice and considering the potential impacts of policy choices on the whole population. By striking this balance, health leaders and the governments they serve can maintain the integrity of the democratic system and the effectiveness of public health policy-making.
